# rapidGSEA: Speeding up gene set enrichment analysis on multi-core CPUs and CUDA-enabled GPUs

**DOI:** 10.1186/s12859-016-1244-x

**Published:** 2016-09-23

**Authors:** Christian Hundt, Andreas Hildebrandt, Bertil Schmidt

**Affiliations:** Department of Computer Science, Johannes Gutenberg University, Staudingerweg 9, Mainz, 55128 Germany

**Keywords:** CUDA, Gene set enrichment analysis, Gene expression data, Resampling statistics

## Abstract

**Background:**

Gene Set Enrichment Analysis (GSEA) is a popular method to reveal significant dependencies between predefined sets of gene symbols and observed phenotypes by evaluating the deviation of gene expression values between cases and controls. An established measure of inter-class deviation, the enrichment score, is usually computed using a weighted running sum statistic over the whole set of gene symbols. Due to the lack of analytic expressions the significance of enrichment scores is determined using a non-parametric estimation of their null distribution by permuting the phenotype labels of the probed patients. Accordingly, GSEA is a time-consuming task due to the large number of required permutations to accurately estimate the nominal *p*-value – a circumstance that is even more pronounced during multiple hypothesis testing since its estimate is lower-bounded by the inverse number of samples in permutation space.

**Results:**

We present rapidGSEA – a software suite consisting of two tools for facilitating permutation-based GSEA: cudaGSEA and ompGSEA. cudaGSEA is a CUDA-accelerated tool using fine-grained parallelization schemes on massively parallel architectures while ompGSEA is a coarse-grained multi-threaded tool for multi-core CPUs. Nominal *p*-value estimation of 4,725 gene sets on a data set consisting of 20,639 unique gene symbols and 200 patients (183 cases + 17 controls) each probing one million permutations takes 19 hours on a Xeon CPU and less than one hour on a GeForce Titan X GPU while the established GSEA tool from the Broad Institute (broadGSEA) takes roughly 13 days.

**Conclusion:**

cudaGSEA outperforms broadGSEA by around two orders-of-magnitude on a single Tesla K40c or GeForce Titan X GPU. ompGSEA provides around one order-of-magnitude speedup to broadGSEA on a standard Xeon CPU. The rapidGSEA suite is open-source software and can be downloaded at https://github.com/gravitino/cudaGSEAas standalone application or package for the R framework.

## Background

High-throughput technologies such as microarray or next-generation sequencing enable researchers to routinely measure the expressions of tens of thousands of genes in many patients. Typically, long lists of interesting candidate genes are generated by subsequent computational analyses. However, interpreting these gene lists is challenging. Recognizing that genes act in concert to drive various biological processes, Gene Set Enrichment Analysis (GSEA) was introduced [1] to summarize genomics data using a predefined gene set. Nowadays, GSEA is a heavily used tool in bioinformatics [2] and has been successfully applied to gain insights into the biological function of diseases such as cancer and diabetes.

However, the GSEA procedure can be highly time-consuming since significance of a calculated enrichment score is typically tested using a resampling strategy drawing large numbers of permutations. When a whole database of gene sets is used, the amount of required permutations is even higher in order to account for multiple hypothesis testing. Furthermore, size and availability of input data sets continue to increase driven by advances in high-throughput technologies [3]. Thus, developing fast software solutions is of high importance to research. Previous work on accelerating gene set analysis has been limited to cloud computing [4]. We present the rapidGSEA suite – an efficient parallelization of the GSEA method for commonly available multi-core CPUs and CUDA-enabled GPUs. By using a combination of parallelization techniques we can achieve speedups of one order-of-magnitude on Xeon CPUs and around two orders-of-magnitude on a single GPU compared to broadGSEA.

## Implementation

This section is divided into three parts. First, we give a brief explanation of the sequential GSEA algorithm and its four major processing steps for estimating the nominal *p*-value of a determined enrichment score using a single gene set. Second, we introduce novel parallelization schemes for single and multiple gene set probing and their explicit implementation optimized for multi-core CPUs and CUDA-enabled GPUs. Finally, we describe the usage of our standalone application and the bundled package for the R framework.

### The sequential algorithm

The traditional GSEA algorithm operates on a real-valued gene expression matrix *D*(*g*_*i*_,*p*_*j*_) of shape |*G*|×|*P*| where *g*_*i*_∈*G* denotes |*G*| unique gene identifiers and *p*_*j*_∈*P* enumerates |*P*| patient identifiers each labelled by a binary phenotype *L*(*p*_*j*_)∈{0,1} encoding cases and controls. The computation of the enrichment score statistics can be split into four major stages:

#### Computation of local deviation measures

For each gene symbol *g*_*i*_ (each row of *D*) a local deviation score *Δ*(*g*_*i*_) is computed that encodes the inter-class deviation between cases and controls. As an example, the difference of means between both classes can be employed to express their variability per gene:

$$\begin{array}{*{20}l} \Delta(g_{i}) &= \mu_{i}^{(1)} - \mu_{i}^{(0)} \\ \mu_{i}^{(1)} &= \sum\limits_{j=0}^{|P|-1} \frac{L(p_{j})}{m^{(1)}} D(g_{i}, p_{j})\\ \mu_{i}^{(0)} &= \sum\limits_{j=0}^{|P|-1} \frac{1-L(p_{j})}{m^{(0)}} D(g_{i}, p_{j}) \end{array} $$

where $m^{(1)} = \sum _{j=0}^{|P|-1} L(p_{j})$ and *m*^(0)^=|*P*|−*m*^(1)^ denote the number of patients in each class from the set {0,1}. Variations that combine intra-class means and standard deviations e.g.

1 $$ \begin{aligned} \begin{array}{lll} \text{fold change:} &\Delta(g_{i}) =\frac{\mu_{i}^{(1)}-\mu_{i}^{(0)}}{\sigma_{i}^{(1)}+\sigma_{i}^{(0)}} &\text{,} \\ \text{t-test:} &\Delta(g_{i}) = \frac{\mu_{i}^{(1)}-\mu_{i}^{(0)}}{\sqrt{\left(\sigma_{i}^{(1)}\right)^{2}+\left(\sigma_{i}^{(0)}\right)^{2}}}& \end{array} \end{aligned}  $$

are common choices for *Δ* in GSEA implementations. Please note that extensions from binary to real-valued phenotype profiles $L(p_{j}) \in \mathbb {R}$ using Euclidean distance, Pearson’s product-moment or Spearman’s rank-order correlation coefficient are straightforward [1] and thus will not be discussed further in this paper.

#### Gene ranking

**Fig. 1:**
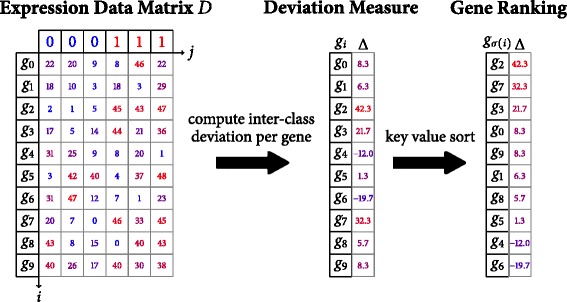
Computation Scheme of Stages 1 and 2. Schematic overview depicting the computation of gene transcription differences (stage 1) and the gene ranking procedure (stage 2) of the sequential GSEA algorithm operation on a single set of phenotype labels (*L*(*p*_*j*_))_*j*_=(0,0,0,1,1,1)

After computation of the local deviations, the indices *i*∈{0,…,|*G*|−1} enumerating the gene symbols *g*_*i*_ are reordered such that

$$\begin{array}{*{20}l} \left(\Delta\left(g_{\sigma(0)}\right), \dots, \Delta\left(g_{\sigma(i)}\right), \dots\Delta\left(g_{\sigma(|G|-1)}\right)\right) \end{array} $$

is a sorted (usually descending) sequence of local deviation scores. The sequence of reordered gene symbols *g*_*σ*(*i*)_ is called *gene ranking* according to *Δ* and will later be used to determine the enrichment score statistic. Figure 1 illustrates the first and second stage of the GSEA algorithm.

#### Enrichment score computation

To elucidate significant differences in gene regulation across different phenotypes, it is generally insufficient to consider transcription differences *Δ*(*g*_*σ*(*i*)_) individually. Each gene can be significantly up- or down regulated by chance alone, or through correlation with processes such as the cell cycle. In principle, information can be gained from clustering genes according to their regulation [5]. Interpretation of the resulting clusters, however, is often unclear. Instead, prior information about gene classes that are assumed to behave correlatedly (e.g. genes on a regulatory pathway), is used in the analysis. Today, this is typically achieved through the framework of GSEA, which considers the significance of the transcription profile of a set of gene symbols *S*⊂*G* as a whole as opposed to individual enrichment values.

**Fig. 2:**
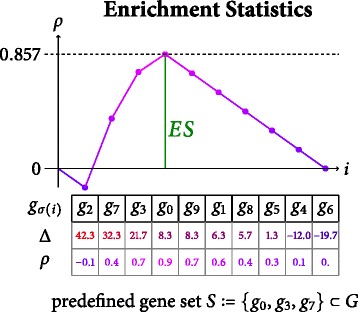
Computation Scheme of Stage 3. Schematic overview of the incremental computation of a linearly-weighted (*p*=1) Kolmogorov-Smirnov statistic operating on our toy data set. The enrichment score is determined by the maximum amplitude of the running sum *ρ*(*k*)

Let *S* be a gene set supposedly correlated to the observed phenotypes and *σ*(*i*) the aforementioned reordering of gene symbols. The *enrichment scoreES*(*S*) is then determined as the maximal amplitude of a weighted running sum statistic *ρ*(*k*)∈[−1,1]:

$$\begin{array}{*{20}l} ES(S) &= \rho\left(\mathop{\text{argmax}}_{k} |\rho(k)| \right) \ \ \quad\text{where} \\ \rho(k) &= \sum\limits_{i=0}^{k} \left\{ \begin{array}{lll} \frac{1}{\alpha} \cdot |\Delta(g_{\sigma(i)})|^{q} & \text{if} & g_{\sigma(i)} \in S \\ - \frac{1}{\beta} & \text{if} & g_{\sigma(i)} \notin S \end{array} \right. \end{array} $$

with precomputed constants $\alpha = \sum _{g \in S} |\Delta (g)|^{q}$ and *β*=|*G*|−|*S*|. The exponent *q*≥0 is usually chosen from the set $\{0, 1, \tfrac {3}{2}, 2\}$ and controls the leverage of the weights |*Δ*(*g*_*σ*(*i*)_)|. Please note that the special case *q*=0 is the well-known Kolmogorov-Smirnov statistic [1]. Figure 2 illustrates an example for the linear-weighted (*q*=1) computation of *ES*(*S*) using a toy data set.

#### Significance estimation

Similar to Pearson’s correlation coefficient the enrichment score takes values in the interval [−1,1] with |*ES*(*S*)|=1 indicating perfect (anti-)correlation and |*ES*(*S*)|≈0 implying no dependency between *S* and the observed phenotypes in terms of the used deviation measure. When *ES*(*S*)=±1 all gene symbols *g*∈*S* are situated at the top/bottom of the ranked gene list. In contrast, small values are observed if the gene symbols *g*∈*S* are scattered over the index domain and thus are unlikely to explain the phenotype distribution.

*ES* values have no intrinsic significance, though. A value of *ES*(*S*)=0.857, as computed in our toy model in Fig. 2, might correspond to a high or low significance, depending on the probability to arrive at such a value by chance alone. Unfortunately, closed forms for the statistical distribution of enrichment score are inaccessible. Therefore, *p*-values are typically estimated by sampling the null distribution using a permutation of phenotype labels. Please note that while some GSEA implementations allow to permute gene identifiers instead of phenotype labels [1, 6] to estimate the null distribution, phenotype permutation is often considered the more appropriate choice – genes are expected to feature statistical dependencies within a single patient, while probes gained from distinct patients are less likely to do so. Hence, in the following we only consider phenotype permutation.

**Fig. 3:**
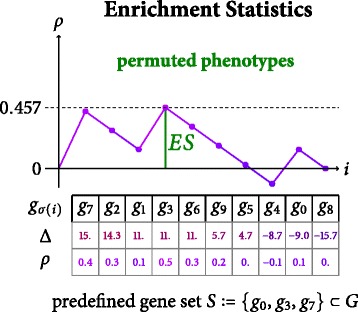
Computation of Enrichment Scores on Permuted Phenotype Labels. Schematic overview of the incremental computation of the enrichment score statistics operating on our toy data set where the phenotypes of patient 1 and 4 have been swapped: $\bigl (L(p_{\pi ^{(1)}(j)})\bigr)_{j} = (0, 1, 0, 1, 0, 1)$

Figure 3 depicts the enrichment score computation for a permutation *π*=(1 4) of the original list of six patients where the columns 1 and 4 of *D* have been swapped.^1^ The resulting score *ES*(*S*,*π*)=0.457<0.857=*ES*(*S*) suggests that the original value is considerably higher than a randomly sampled one. An exact computation of the *p*-value – due to absent closed forms for their distribution – would require us to calculate *ES*(*S*,*π*) for all |*P*|! permutations and finally determine the portion of values which are more extreme than *ES*(*S*). GSEA implementations hence usually estimate *p*-values by sampling in the space of permutations since |*P*|! is too large even for a moderate number of patients.

When probing more than one gene set at once, *p*-value estimates have to be adjusted for multiple hypothesis testing. As an example, Bonferroni-corrected acceptance levels and family-wise error rates (FWER) are frequently used criteria to evaluate the significance of enrichment scores. The need for a large number of samples in the space of permutation is even more pronounced during multiple hypothesis testing: let *e*∈*Π* be the identity permutation in the set of *n* tested permutations *Π*. Then the *p*-value estimate for a fixed gene set *S* is strictly positive [7] and lower-bounded by inverse sample size:

$$\begin{array}{*{20}l} \hat p_{S} = \frac{1}{n}\sum\limits_{\pi\in\Pi} \left(|ES(S, \pi)| \geq |ES(S, e)| \right) \geq \frac{1}{n} \end{array} $$

The Molecular Signature Database v5.1 [8] contains more than 13,000 gene sets divided into eight major collections. Thus, when testing all gene sets at a Bonferroni-adjusted significance level of $\alpha = \frac {0.01}{13,000}$ we have to probe more than 1,300,000 permutations in order to allow the result $\hat p_{S} < \alpha $. For the rest of the paper, we focus on the efficient computation of the enrichment score table *ES*(*S*,*π*) since *p*-value estimates and other statistics such as FWER can be determined using its entries in a post-processing phase.

### The parallel algorithm

GSEA can be parallelized using coarse-grained computation schemes such as assigning threads to each permutation *π* or gene set *S* since all entries in *ES*(*S*,*π*) can be processed independently. This approach will be used in our multi-threaded shared memory implementation of GSEA (ompGSEA): The set of *n* probed permutations is split into *m* partitions each of approximate size $\frac {n}{m}$ and afterwards *m* threads independently operate on the individual chunks. This can easily be achieved in shared memory architectures using OpenMP pragmas. Moreover, extensions to distributed memory architectures using the Message Passing Interface (MPI) are conceivable.

However, CUDA-enabled accelerators can maintain up to several thousands of threads (e.g. Titan X/Tesla K40c: 3,072/2,880 cores) but only exhibit a limited amount of RAM (both GPUs provide 12 GB). As a result, fine-grained computation schemes that parallelize the aforementioned building blocks of the GSEA algorithm have to be employed to exploit the full compute capabilities of CUDA-enabled accelerators. In the following, we will present the fine-grained parallelization scheme for each processing stage separately.

#### Computation of local deviation measures

Many local deviation measures used in traditional GSEA e.g. *difference of means* or *fold change* can be expressed in terms of intra-class means and standard deviations. Therefore, we have to separately accumulate sums of expression values and their squares for each of the two phenotypes. Although efficient implementations for parallel reduction on CUDA-enabled accelerators are known [9] we instead parallelize the loop over the gene symbols since each row of the data matrix *D* can be processed independently without the need for expensive synchronization as used in reduction algorithms. Moreover, the number of gene symbols will most likely exceed the number of probed patients and thus the loop over *g*_*i*_ is better suited for massively parallel computation. During the calculation of statistical moments we encounter two challenges:

First, the numerically stable computation of standard deviations is known to be a stubborn task. On the one hand, when accumulating a large number of entries (here patients) one has to account for numeric stability using cancellation-compensation [10] or two-pass algorithms for the standard deviations. On the other hand, when dealing with only a few patients one-pass or cancellation-compensated online algorithms for the standard deviation might be a proper trade-off between accuracy and speed [11]. rapidGSEA exploits the C++ template engine to provide specialized and user-customizable accumulator functors adaptable to the task’s requirements.

**Fig. 4:**
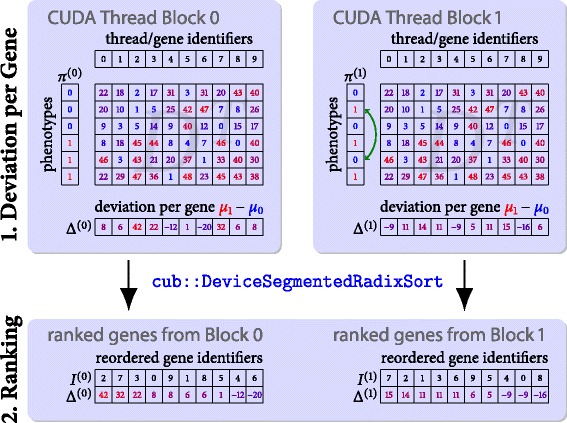
Fine-Grained Parallelization of Stages 1 and 2. Parallelization of the deviation score computation operating on the transposed data matrix *D* ^*T*^. Each thread block draws a permutation by shuffling the original phenotype label list in shared memory. The threads within a thread block independently accumulate gene transcription differences for each gene symbol identifier (along columns) ensuring coalesced reads from global memory. Finally, the local deviation scores are sorted using the segmented radix sort primitive of CUB

Second, the gene-wise computation of transcription differences *Δ*(*g*_*i*_) accumulates statistical moments along the rows of the matrix *D*. Using a CUDA thread block of up to 1,024 CUDA threads for a fixed permutation of the phenotype array *L*(*p*_*π*(*j*)_) it is advisable to transpose *D* to guarantee coalesced access to global memory. More specifically, since a warp of 32 threads is executed simultaneously on the GPU their concurrent reads from the same column of *D* would result in excessive cache misses. In contrast, when transposing *D* the same access pattern causes consecutive threads to simultaneously access consecutive memory. This change from column-major-order to row-major-order traversal decreases the runtime of this processing step by one order-of-magnitude in our experiments. Since *D* usually tends to be smaller than 100 MB, we can use a standard bank conflict-free out-of-place algorithm for matrix transposition [12]. Figure 4 depicts the described computation scheme for two CUDA thread blocks each consisting of ten CUDA threads. Please note that the genes are distributed using a block-cyclic distribution if the number of genes exceed the number of threads.

The sampling of permutations can be accomplished using the pseudo random number generators (PRNG) from the cuRAND library [13] bundled with the CUDA SDK. Unfortunately, cuRAND does not provide host-sided calls for the random number generators defined in the device API. Thus, we implemented the *keep it simple stupid* (KISS) PRNG [14] for the CPU and GPU in order to provide consistent results across architectures. Both cuRAND’s *xorwow* PRNG and our KISS implementation pass all tests of the dieharder suite [15]. The permutation of the phenotype labels *L*(*p*_*π*(*j*)_) is generated by reordering the original label list *L*(*p*_*j*_) in shared memory using a Fisher-Yates shuffle.

#### Gene ranking

Up to this point, the transcription differences *Δ*(*g*_*i*_) have been computed for a batch of permutations that fit into the RAM of the GPU. Unfortunately, we cannot directly apply a key-value sort to *Δ*(*g*_*i*_) within the same kernel due to the 48 KB limitation of shared memory. Thus, after termination of the previous kernel, we call a device-wide key-value radix sort primitive cub::DeviceSegmentedRadixSort from the CUB [9] library specifically optimized for the efficient sorting of segmented arrays. This approach is up to one order-of-magnitude faster than stacking single device-wide cub::DeviceRadixSort calls for each permutation or aliasing global memory to the block-wide cub::BlockRadixSort primitives. The number of concurrently sorted arrays has been set to 128 as a proper trade-off between runtime and memory consumption. At the end of this stage, we have stored the sorted deviation scores *Δ*(*g*_*σ*(*i*)_) and corresponding indices *σ*(*i*) for each of the probed permutations in global memory. Figure 4 illustrates the described workflow.

#### Enrichment score computation

The computation scheme for the running sum statistic is similar to the processing of local deviation scores. For each permutation a CUDA thread block operates on a pair (*g*_*σ*(*i*)_,*Δ*(*g*_*σ*(*i*)_)) of reordered gene symbols and gene transcription differences. The test whether a gene identifier is part of a gene set *g*_*σ*(*i*)_∈*S* is usually implemented with hash sets on CPUs. Efficient hashing algorithms on CUDA-enabled devices are stated in the literature [16] which typically involve linked lists or binary search in sorted arrays in order to resolve collisions. However, we decided to encode the affiliation of a gene *g* with a binary bit mask *b*(*g*,*S*). The computation of the bit mask can be delegated to the CPU using STL hashes. Further, the corresponding execution time can be overlapped with the deviation score and gene ranking kernels. As a result, we can determine a gene’s affiliation on the GPU in constant time by reading the corresponding entry of the bit mask from global memory.

**Fig. 5:**
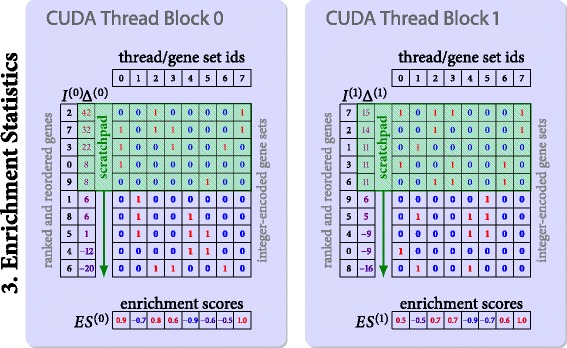
Fine-Grained Parallelization of Stage 3. Parallelization of the enrichment score computation operating on the ranked genes and precomputed bit masks. Again, each thread block processes a permutation. The threads within a thread block independently accumulate the running sum statistic for each of the probed gene sets. Shared memory is utilized to suppress redundant reads from global memory

Each thread *k* within a thread block processes one gene set *S*_*k*_. Shared memory can be utilized to avoid slow accesses to global memory since all threads in a warp have to access the same entry from the bit mask *b*(*g*_*σ*(*i*)_,*S*_*k*_) in random order. To achieve this, batches of 64 entries of reordered gene transcription differences *Δ*(*g*_*σ*(*i*)_) and bit mask entries *b*(*g*_*σ*(*i*)_,*S*_*k*_) are consecutively loaded into shared memory (scratchpad) and afterwards processed in order. Due to the large number of genes we again use numerically stable Kahan summation [10] in order to suppress cancellation in floating point arithmetic. Finally, the maximum amplitude of the weighted Kolmogorov-Smirnov statistic is written to the enrichment score table *ES*(*S*,*π*) and consecutively transferred to the host. Figure 5 illustrates the described procedure.

#### Significance estimation

When only computing *p*-value estimates the counting of values in the tails of the null distribution could be accomplished on the GPU using the device-wide reduction primitive cub::DeviceSegmentedReduce from the CUB library. A similar approach for the computation of the FWER is conceivable. However, we decided to copy *ES*(*S*,*π*) to the host in order to provide the full information for consecutive analysis and visualization of sampled distributions.

### Bindings for the R language

The core algorithm written in CUDA and C++11 is provided as standalone application and additionally as Rcpp-based [17] package for R. The latter includes functions for the reading of gene expression tables (*.gct), class assignment labels (*.cls) and gene sets files (*.gmt) as well as methods for the querying and selection of the used GPU (see user manual).

## Results and discussion

The performance of rapidGSEA is compared to the broadGSEA Java application in version 2.2.2 [18] on the following platform:

- **(CPU)** Intel Xeon E5-2660 v3 @ 2.60 GHz GHz (10+10 HT) with 128 GB DDR4 RAM
- **(GPU)** NVIDIA GeForce GTX Titan X with 12 GB GDDR5 RAM, NVIDIA Tesla K40c with 12 GB GDDR5 RAM disabled ECC, NVCC ver. 7.5
- **(Software)** Ubuntu 14.04 LTS, GCC ver. 4.8.4, IcedTea ver. 2.6.3 OpenJDK 64-Bit Server VM

In our experiments, we use gene expression data (GEO: Series GSE19429) consisting of 183 MDS patients and 17 healthy controls where the array spots have been collapsed to |*G*|=20,639 unique gene symbols by max pooling ambiguous mappings in the Affymetrix Human Genome U133 Plus 2.0 Array (GEO: Platform GPL570) [19]. We further choose the smallest (H: hallmark, 50 gene sets) and the biggest (C: curated, 4726 gene sets) collection from the Molecular Signatures Database 5.0 [8]. The number of tested permutations ranges from 1,024 up to 1,024^2^ = 1,048,576 samples. Single-precision runs are executed on the GeForce GTX Titan X and double-precision experiments on the Tesla K40c GPU. If not stated otherwise, rapidGSEA and broadGSEA have been configured to read the input data from disk and afterwards to write the full enrichment score table *ES*(*S*,*π*) to the file system in order to ensure fair competition.

### Accuracy and compliance of enrichment scores

**Fig. 6:**
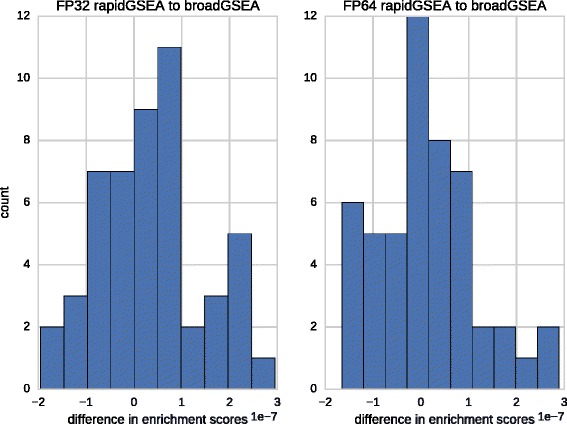
Compliance of computed enrichment scores. Histograms of the difference of computed enrichment scores between rapidGSEA and broadGSEA over the 50 gensets from the Hallmark collection. Both, single and double-precision residues comply within six digits

We have evaluated the compliance of computed enrichment scores between broadGSEA and rapidGSEA using the identity permutation on the 50 gene sets of the Hallmark collection under the *difference of classes* measure. The deviation of computed enrichment scores between rapidGSEA and broadGSEA comply within six digits for both single and double-precision arithmetic (see Fig. 6). Using identical floating point data types the computed scores of both rapidGSEA components, cudaGSEA and ompGSEA, are indistinguishable.

**Fig. 7:**
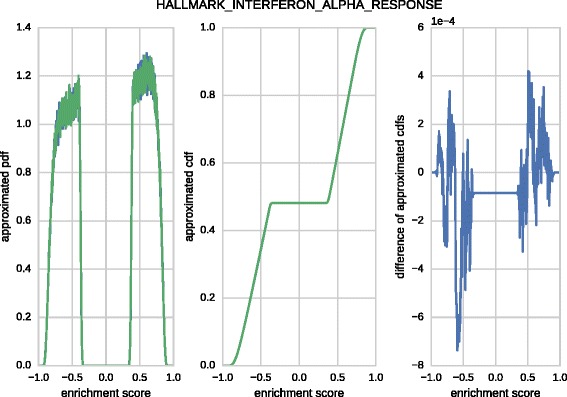
Compliance of computed histograms of enrichment scores. Histograms of the computed enrichment scores of rapidGSEA and broadGSEA using *n*=1,024^2^ permutations over one of the 50 gensets from the Hallmark collection. Both, the computed PDFs (*left* panel) and CDFs (*middle* panel) are visually almost indistinguishable. The absolute difference of CDFs (right panel) and thus the absolute error of *p*-values is bounded by less then 0.001 in this example

However, a comparison of computed histograms *ES*(*S*,*π*) is more complex due to different implementations of random number generators. Thus, we have approximated the probability density functions (PDFs) of the enrichment score distribution using *n*=1,024^2^ permutations and $\sqrt {n} = 1,024$ bins uniformly sampling the interval [−1,1]. Afterwards, the approximate cumulative distribution functions (CDFs) are computed by prefix summation. The maximum absolute difference of approximated CDFs, also know as Kolmogorov distance,

$$\begin{array}{*{20}l} dist = \max\limits_{k} | CDF^{\mathrm{(rapidGSEA)}}_{k} - CDF^{\mathrm{(broadGSEA)}}_{k} | \end{array} $$

is then determined for each of the 50 gene sets. Note, the Kolmogorov distance is a reasonable choice since it determines the measurement error of the area under the PDF of the enrichment score distribution and thus relates to the error of the estimated *p*-value. Figure 7 visualizes the described procedure for one gene set. The minimum/median/maximum absolute deviation between the approximated CDFs produced by rapidGSEA and broadGSEA over the 50 gene sets is given by 0.0005/0.0011/0.0018. When comparing two histograms both computed by broadGSEA with different seeds the same metrics yield 0.0006/0.0011/0.0018. Moreover, in 26 out of 50 cases rapidGSEA produces histograms with a smaller Kolmogorov distances to broadGSEA in contrast to 24 cases where both histograms produced by broadGSEA are more similar. Concluding, the deviations in estimated areas are reasonably small and mainly caused by different samples in permutation space.^2^

### Scaling over multiple cores

**Fig. 8:**
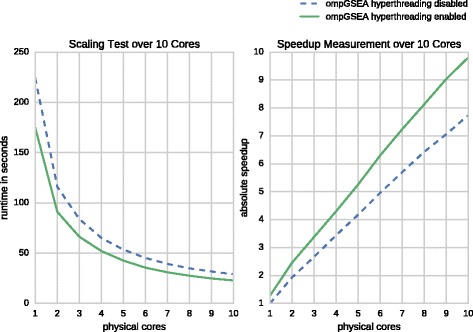
Scaling ompGSEA over multiple cores. Runtime in seconds (*left* panel) and speedup in comparison to single-threaded performance (*right* panel) using up to ten physical cores with and without hyperthreading

We perform a strong scalability test of our ompGSEA implementation over multiple cores of the Xeon CPU. Note, ompGSEA is part of the cudaGSEA binary and can be selected using the -cpu flag. The time needed to process the 50 gene sets defined in the H(allmark) collection is measured for a fixed input size of *n*=16,384 permutations and a variable number of threads. The experiments cover performance measurements for up to ten physical cores each executing a single thread and a hyper-threaded scenario where up to twenty threads are assigned to ten physical cores. When taking measurements on less than ten physical cores we enforce a thread’s affinity using the taskset command in order to avoid rescheduling by the operating system. The obtained runtimes are listed in Table 1 and illustrated in Fig. 8. The first experiment utilizing only physical cores reveals almost linear speedup for ompGSEA with an efficiency of roughly 77 *%* for ten cores. However, the hyper-threaded variant exhibits slightly super-linear behaviour for up to nine physical cores and an efficiency of 98 *%* for all cores. Throughout the rest of this paper all reported runtimes of ompGSEA refer to the hyper-threaded ten core scenario running approximately ten times faster than the corresponding single-core application. Please note that the time for writing the enrichment score table *ES*(*S*,*π*) to disk has been neglected during this benchmark.

**Table 1:** Scaling over multiple cores

**noHT**	**1**	**2**	**3**	**4**	**5**	**6**	**7**	**8**	**9**	**10**
Runtime	224.11	116.06	83.93	65.11	53.56	45.21	39.31	34.87	31.74	28.94
Speedup	1.00	1.93	2.67	3.44	4.18	4.96	5.70	6.43	7.06	7.74
Efficiency	1.00	0.97	0.89	0.86	0.84	0.83	0.81	0.80	0.78	0.77
**HT**	**1**	**2**	**3**	**4**	**5**	**6**	**7**	**8**	**9**	**10**
Runtime	174.82	91.09	66.31	52.09	42.57	35.58	30.94	27.55	24.79	22.85
Speedup	1.28	2.46	3.38	4.30	5.26	6.30	7.24	8.13	9.04	9.81
Efficiency	1.28	1.23	1.13	1.08	1.05	1.05	1.03	1.02	1.00	0.98

Runtime in seconds, speedup and parallelization efficiency using up to ten physical cores with disabled hyperthreading (noHT) and enabled hyperthreading (HT) for a fixed number of *n*=16,384 permutations on the Hallmark gene set collection

### Comparison between rapidGSEA and broadGSEA

**Fig. 9:**
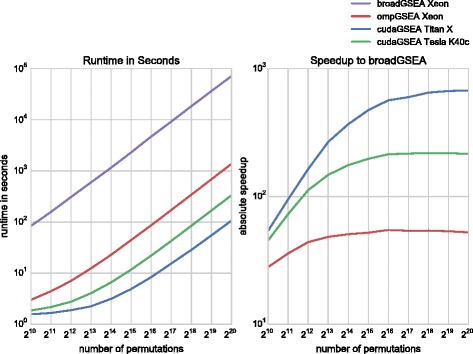
Performance Comparison between rapidGSEA and broadGSEA on Hallmark Gene Set Collection (H). Runtime in seconds of rapidGSEA and broadGSEA (*left* panel) and speedups of rapidGSEA in comparison to broadGSEA (*right* panel) for up to 1,024^2^ permutations on the Hallmark (H) collection consisting of 50 gene sets

**Fig. 10:**
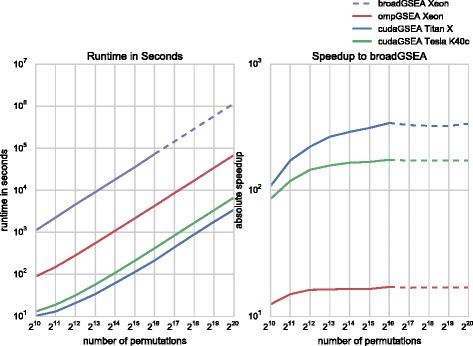
Performance Comparison between rapidGSEA and broadGSEA on Curated Gene Set Collection (C2). Runtime in seconds of rapidGSEA and broadGSEA (*left* panel) and speedups of rapidGSEA in comparison to broadGSEA (*right* panel) for up to 1,024^2^ permutations on the Curated (C2) collection consisting of 4,725 gene sets. Please note that dashed lines indicate linearly extrapolated results in log-log space

The execution time of rapidGSEA and broadGSEA is measured on the aforementioned data set over a wide range of permutations (1,024 up to 1,024^2^) using the Hallmark (H: 50 gene sets) and Curated (C2: 4,725 gene sets) collections. The experiments include parsing of input files, memory transfers over PCIe when using CUDA and writing the enrichment score table *ES*(*S*,*π*) to spinning disk. The obtained runtimes and speedups are listed in Table 2 and illustrated in Figs. 9 and 10. Numbers in square brackets or dashed lines indicate linearly extrapolated runtimes for broadGSEA in log-log space for large amounts of permutations.

**Table 2:** Performance comparison between rapidGSEA and broadGSEA

**H (50)**	**1,024**		**4,096**		**16,384**		**65,536**		**262,144**		**1,048,576**	
broad Xeon	83.6		307.1		1,149.0		4,681.0		18,301.0		70,946.8	
rapid Xeon	3.0	(28)	7.0	(44)	22.7	(51)	85.8	(55)	339.4	(54)	1,357.8	(52)
rapid Tesla	1.8	(45)	2.7	(112)	6.5	(176)	21.9	(214)	83.7	(219)	329.9	(215)
rapid Titan	1.6	(54)	1.9	(164)	3.1	(367)	8.3	(564)	28.2	(648)	105.2	(674)
**C2 (4,725)**	**1,024**		**4,096**		**16,384**		**65,536**		**262,144**		**1,048,576**	
broad Xeon	1,113.0		4,600.0		17,962.0		70,213.4		[274,464.0]		[1,072,878.9]	
rapid Xeon	89.3	(12)	282.9	(16)	1,084.8	(17)	4,266.2	(16)	17,069.0	(16)	68,682.3	(16)
rapid Tesla	13.1	(85)	31.6	(146)	108.5	(166)	418.7	(168)	1,685.5	(163)	6,732.6	(159)
rapid Titan	10.3	(108)	20.8	(221)	61.9	(290)	214.0	(328)	895.9	(306)	3,447.3	(311)

Runtime in seconds and speedups of rapidGSEA compared to broadGSEA (round brackets) using up to *n*=1,024^2^ permutations on the Hallmark (H: 50 gene sets) and Curated (C2: 4725 gene sets) collection. Please note that runtimes in square brackets indicate linearly extrapolated runtimes of broadGSEA in log-log space

Our multi-threaded implementation ompGSEA outperforms broadGSEA on both gene set collections (H and C2) by at least one order-of-magnitude. Note, although broadGSEA spawns more than twenty threads the majority remains idle during processing. Therefore, broadGSEA cannot benefit from the additional physical cores of the Xeon processor. The same behaviour can be observed on an Intel i7 i3970X CPU with six physical cores.

Moreover, cudaGSEA outperforms broadGSEA by around two orders-of-magnitude with growing speedups for an increasing number of permutations. This can be explained by the thread occupancy of the used GPUs. Both, the GeForce Titan X and the Tesla K40c can store at once tens of thousands of permutations (roughly 70k/35k in single/double-precision) within their 12 GB of RAM. Thus, when probing a small number of permutations the majority of streaming multi-processors remain idle. Furthermore, the parsing of input files and dumping of results takes several seconds and cannot be parallelized on the GPU.

## Conclusions

In this paper, we have introduced rapidGSEA – a software suite consisting of two tools for facilitating permutation-based GSEA: cudaGSEA and ompGSEA. cudaGSEA is a CUDA-accelerated tool using fine-grained parallelization schemes on massively parallel architectures while ompGSEA is a coarse-grained multi-threaded tool for multi-core CPUs. ompGSEA outperforms the state-of-the-art implementation of GSEA (broadGSEA) by at least one order-of-magnitude in terms of execution times while providing compliant results. Furthermore, cudaGSEA outperforms broadGSEA by around two orders-of-magnitude. The time for probing 1,048,576 permutations on a gene expression data set consisting of 20,639 unique gene symbols and 200 patients can drastically be reduced from roughly 13 days for broadGSEA to less than two hours using rapidGSEA on a commonly available Tesla K40c GPU in double-precision or less than one hour on a GeForce Titan X in single-precision.

A possible direction of future research in order to further reduce runtimes is the parallelization of GSEA on a compute cluster with multiple GPUs attached to each node. Furthermore, extensions of GSEA to consider graph-based (Gene Graph Enrichment Analysis [20]) or network-based (Network-based GSEA [21]) correlations between gene symbols and observed phenotypes have gained increasing attention in recent years. It will be interesting to investigate how the parallelization techniques discussed in this paper can be applied to accelerate these extended enrichment methods.

## Availability and requirements

**Project name:** cudaGSEA **Project home page:** https://github.com/gravitino/cudaGSEA**Operating system(s):** Linux **Programming language:** C++, CUDA, R **Other requirements:** CUDA-capable GPU**License:** GNU LGPL **Any restrictions to use by non-academics:** None

## Endnotes

^1^ Please note that throughout this manuscript, we use zero-based indexing.

^2^ Individual results for each gene set can be found at the github repository of rapidGSEA.

## Abbreviations

API: Application programming interface
CUDA: Compute unified device architecture
FWER: Family-wise error rate
GSEA: Gene set enrichment analysis
MPI: Message passing interface
PCIe: Peripheral component interconnect express
PRNG: Pseudo random number generator
